# Menstrual health and menstrual equity for women living with HIV: a minireview

**DOI:** 10.3389/frph.2025.1580783

**Published:** 2025-09-18

**Authors:** Shilpa Melanie Darivemula, Lisa Rahangdale

**Affiliations:** ^1^ Division of General Obstetrics and Gynecology, Department of Obstetrics and Gynecology, University of North Carolina at Chapel Hill, Chapel Hill, NC, United States; ^2^ Lineberger Comprehensive Cancer Center, University of North Carolina at Chapel Hill, Chapel Hill, NC, United States; ^3^ Center for AIDS Research, University of North Carolina at Chapel Hill, Chapel Hill, NC, United States

**Keywords:** menstrual equity, menstrual health, stigma, human immunodeficiency virus, gynecology

## Abstract

Despite the advent of ART and the conversion of human immunodeficiency virus (HIV) into a chronic disease, little is known regarding the experiences of women living with HIV (WLWH) in accessing knowledge support and supplies for menstrual health. The inability to access supplies or manage vaginal bleeding safely negatively impacts menstrual equity, a term used to address vaginal bleeding needs across the reproductive life course. For WLWH, these inequities are compounded with additional externalized and internalized stigma, making them especially vulnerable to poor gynecological care utilization and menstrual health management. This review introduces recent research on the nascent topic of menstrual equity in the United States and explores existing data on menstrual health and its intersections with stigma and access in WLWH. The goal of this review is to highlight current evidence and persisting gaps in menstrual health research for WLWH in the United States and emphasizing potential future developments in addressing the common yet hidden issue of menstrual inequity in this population.

## Introduction

In 2022, there were about 268,800 women living with HIV (WLWH) in the United States (1–3). Adequate treatment with antiretroviral therapy (ART) has increased the lifespan of those living with human immunodeficiency virus (HIV) which has allowed for menstruating WLWH to experience the full spectrum of gynecologic transitions over their life course (3, 4). From menarche to the postpartum phase and to menopause, these reproductive transitions are often identified by changes in volume, pain, and frequency of vaginal bleeding (5). Several studies have described the negative impacts of heavy or irregular bleeding on physical markers of health in women, such as worsening anemia, as well as mental markers, such as decreased quality of life (5–8). Additionally, the availability of potent multi-drug ART and knowledge on the concept of U = U (Undetectable = Untransmittable)–that individuals with suppressed HIV viral loads on ART will not transmit to sexual partners–has transformed the transmission risks of vaginal intercourse (9). It is unclear how current patient perceptions of transmission risk, HIV-related stigma, and societal and health inequities impact the experience of menstruation in WLWH.

Research on managing menses with dignity in the United States—otherwise known as menstrual equity—focuses on many socioeconomically and culturally vulnerable populations (Table 1). There is limited information on menstrual needs, support, and access for WLWH—a unique population in which social, economic, racial, and clinical risk factors for menstrual inequities intersect with potential blood stigma and known marginalization. This mini review highlights current gaps in menstrual health and menstrual equity research and describes why addressing menstrual health and equity are essential for WLWH. For the purposes of this review, we will use the term WLWH given its widespread use in current literature while acknowledging that some menstruators may not identify as women.

**Table 1:** Cohort-based menstrual equity studies on unique U.S. Populations.

Authors	Year of publication	Type of study	Population studied	Reproductive phase of life	Associations with menstrual inequity
Sebert Kuhlman, et al. (10)	2019	Survey and Focus Groups	Low-income women and community service members in St. Louis, MO	Women of reproductive age (18–69 years)	Food insecurity, Employment, House-lessness,
Keiser, et al. (11)	2020	Survey	Menstruators with substance use disorder undergoing outpatient treatment in Richmond, VA	Women of reproductive age (18–51 years)	Food insecurity, substance use, economic instability, missed work, distance to purchase products
Lane et al. (12)	2020	Interviews, Online Writing Activity	Trans and non-binary people and clinical staff caring for LBGTQ population in New York City, NY	Male-aligned, transman, transmasculine, non-binary, queer man and FTM (17–32 years)	Bodily sovereignty, poor access to gender supportive care
Secor-Turner et al. (13)	2020	Focus Group Interviews	Adolescents in North Dakota	Adolescents (12–16 years)	Lack of knowledge, Fear around menarche, lack of school support
Cardoso et al. (8)	2021	Surveys	College aged women, national cohort	Young adults (18–24 years)	Depression
Sommer et al. (14)	2021	Surveys	Adults, national cohort	Adults (25–34 years)	Pandemic related income loss, food insecurity, housing insecurity, rurality
Darivemula, et al. (15)	2023	Surveys	Adults involved with the Carceral System, Kansas	Adults	Incarceration
Davies et al. (16)	2023	Interviews	Adolescents, Philadelphia, PA	Adolescents (13–24 years)	Education, financial burden, limited access to supplies
Casola et al. (17)	2024	Interviews	Adults, Philadelphia, PA	Adults (18–45 years)	Menstrual communication stigma in families and clinics
Wolff et al. (18)	2024	Surveys	Adolescents, national	Adolescents (14–24 years)	Limited access to supplies and gynecologists
Darivemula et al. (5)	2024	Video and Audio Recording	Adults, Chapel Hill, North Carolina	Adults (18–35 years old)	Language barriers, financial burden, education, anxiety
DeMaria et al. (19)	2024	Interviews	Adults experiencing homelessness, Indiana and Chicago	Adults experiencing homelessness and service providers	Lack of access to public restrooms, mental health burden, shame and embarrassment (stigma)

This table highlights cohort based studies on menstrual equity in the United States since the advent and popularization of the term “menstrual equity” in 2017. While there are several more studies on those facing homelessness and adolescents in universities, they are not included given their population is already represented below.

## A brief summary of menstrual equity research in the United States

Menstrual equity is defined as access to safe spaces for menstruation, access to menstrual products, and the ability to engage in daily life with dignity and without stigma or discrimination (15). Initially considered a policy term for global improvements in reducing state-level taxation on products and improving access to free supplies in schools (20), recently, increasing cohort-based studies on varying vulnerable populations have focused on community-specific barriers to access, support, and safety in the United States across the reproductive lifespan (Table 1). Period poverty is often used interchangeably with menstrual equity in existing literature (20).

Table 1 outlines cohort-based studies on menstrual equity in unique populations in the United States published in the last five years after the advent of the term “menstrual equity” by Jennifer Weiss-Wolf in 2017 (15). Using search terms such as “menstrual cycle”, “menstruation”, “menarche”, “menstrual hygiene products”, “amenorrhea”, “abnormal uterine bleeding”, “period poverty”, “pink tax”, “menstruating”, “oligomenorrhea”, “heavy menstrual bleeding”, and “equity”, “equities”, “inequity” “inequities”, “disparity”, “disparities”, and “United States”, a total of 63 studies were identified as occurring in the United States in SCOPUS, PubMed, and CINAHL. Studies were excluded if they were not primary cohort studies. Each study was then evaluated for the population of focus, be it adolescents, people without homes, racial and ethnic groups, or gender diverse people. Representative studies of each unique population evaluated for menstrual equity in the United States were included in Table 1, highlighting the limited number of populations studied to determine the already understudied extent and experience of menstrual inequities.

**Figure 1:**
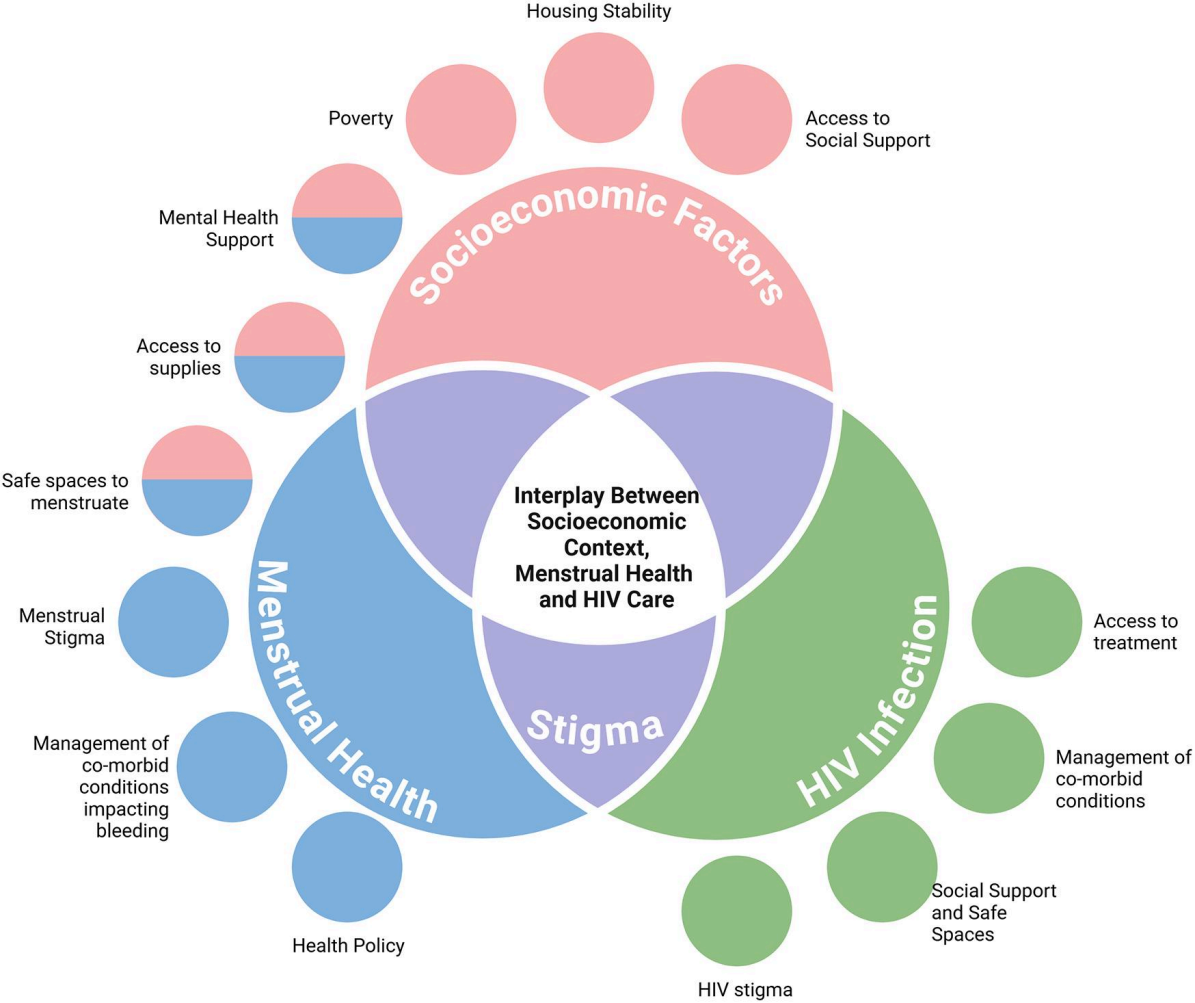
Factors impacting menstrual equity for WLWH. Based on prior studies, several factors impact both menstrual health and living with HIV. Highlighted above are a few of those factors. Stigma is noted as a major overlap between both menstrual health and HIV care. Created in https://BioRender.com.

Much of the existing data on community-specific menstrual equity focuses on adolescents and college-aged youth, specifically emphasizing the relationship between menstrual inequities and mental health (8, 20, 21). There are few menstrual equity studies that focus on specific reproductive phases, such as the postpartum phase (5), and there are no studies on menstrual equity during terminal reproductive transitions, such as the perimenopausal phase. In fact, menstrual stigma persists across every reproductive transition (16); this shame and embarrassment and its nuanced presentation across the reproductive lifespan—a major barrier to clinical communication and treatment access–remains understudied (16, 17). There is a lack of current research on menstrual health and equity for WLWH, despite the many overlapping social, medical, cultural, and financial barriers shared between those living with HIV and those living with menstrual inequities as described in Figure 1 (16–22). For WLWH, the combination of known menstrual irregularities (6), inadequate access to menstrual healthcare utilization (23), intersecting challenges with social determinants of health (24), and blood infectivity stigma (25–27) highlight the need to specifically understand menstrual health and menstrual equity needs in this dually vulnerable population.

## A brief summary of menstrual health research on WLWH

Several studies on menstrual cycle characteristics of WLWH were conducted on mainly global populations in the late 1990s and early 2000s and demonstrated varying results regarding (*1*) the role that HIV infection plays on the HPA axis, (*2*) the types of menstrual irregularities experienced by WLWH, and (*3*) the effects of menstrual burden and blood stigma on reproductive decision-making. Initial screening for studies on HIV and menses resulted in 89 studies globally. Given such a small sample size, the decision was made to evaluate all articles for menstrual health research for WLWH, despite the aim to focus on menstrual equity in WLWH in the United States to emphasize a current and important gap in the literature. No study specifically evaluated menstrual equity or period poverty in this population in the United States or globally. Articles that specifically discussed menstrual bleeding, abnormal uterine bleeding, amenorrhea, oligomenorrhea, menstrual equity, and period poverty in WLWH were included. This resulted in a total of 25 articles for final review, with the following areas of focus.

## HIV infection and the hypothalamic-pituitary-adrenal axis

The mechanisms driving abnormal menstruation in WLWH remain unclear, however, many studies identify several potential theories (6, 22, 28). HIV infection can induce pro-inflammatory cytokine activity, leading to changes in the neuroendocrinological communication of the Hypothalamic-Pituitary-Adrenal Axis and subsequent ovulatory dysfunction (6, 28). In WLWH with ovulatory dysfunction, studies have demonstrated abnormalities with follicle-stimulating hormone, luteinizing hormone, and prolactin (29). While these abnormalities may be from the infection itself, several studies indicate that comorbidities, such as poor nutrition, ongoing intravenous drug use, or low BMI, increase risk of menstrual abnormalities when compared to healthy WLWH (6). No current studies highlight relationships between HIV infection and increased frequency of structural causes of abnormal menstruation, such as polyps, fibroids, or polycystic ovarian syndrome. In fact, a single academic center cohort study in 2005 noted that despite increased visceral adiposity in WLWH, there was no parallel increase in PCOS incidence when compared to healthy controls (30). Additionally, there is no consensus on the effect of ART on the HPA axis (31).

## Characteristics of abnormal menstruation in WLWH

Studies over the past few decades have highlighted a diverse range of abnormal menstruation experienced by WLWH, often with conflicting conclusions (6, 32–34). A 1996 cross sectional study comparing the menstrual cycles of women with and without HIV noted that the abnormal uterine bleeding –defined as presence of post-coital bleeding, intermenstrual bleeding, or amenorrhea—did not significantly differ, even in women with CD4 levels less than 200 cells/microL (32). This study noted that most WLWH had an intact hypothalamic-pituitary-ovarian axis and experienced a 25–35-day menstrual cycle, indicating that other health factors outside of living with HIV impacted menstrual health (32). A 2010 article noted that in WLWH in Nigeria, rates of amenorrhea, oligomenorrhea, and irregular bleeding were higher than in WLWH; these rates, increased with lower CD4 counts and lack of ART use (33). A 2024 review of menstrual irregularities in WLWH in Nigeria, further confirmed the role of external factors beyond HIV impacting menstrual health, noting usually menstrual irregularities were due to secondary comorbid conditions such as low weight, nutritional deficiencies, or HIV-induced cytopenia, rather than direct HPA axis impact by the virus (24).

Studies in the United States noted little overall effect of HIV serostatus directly on amenorrhea, menstrual cycle duration, and menstrual cycle variation (22, 35). Studies in the early 2000s highlighted the incidence of secondary amenorrhea as a major patient concern in WLWH. Several studies describing this increased incidence of amenorrhea note that potential hormonal dysfunction could be secondary to comorbid exposures such as drug use, anorexia, chronic disease, or weight fluctuations (32). Prolonged secondary amenorrhea was described in several studies with WLWH with higher BMI and in those who are not adherent with ART (22, 35). The pharmacological effects of ART on menstrual cycles remains unclear, but early cross-sectional studies do not highlight any associations (6). WLWH more often than women without HIV can experience prolonged amenorrhea and experience missed opportunities for treatment of metabolic abnormalities, evaluation of infertility, or accessing hormone replacement therapy in a timely fashion (4, 22, 32).

While many studies explored the relationship between secondary amenorrhea in WLWH, a large national cohort study highlighted that nearly one-third of WLWH experienced heavy menstrual bleeding (6). Factors associated with abnormal menstruation included ART use, hepatitis B co-infection and tobacco use (6). Another study from this cohort reported over 50% of WLWH stated they used contraception to regulate menstrual periods (36).

Despite the documented wide prevalence of abnormal bleeding in this population in the 1990s and 2000s, there are no studies in the past 10 years that address updates in existing menstrual abnormalities, and etiologies in this population –information that may have changed given the increased availability and uptake of newer highly active ART regimens and improvements in HIV-related care.

## Effects of menstrual burden and potential blood stigma

HIV-related stigma is defined as “the shame or disgrace attached to this disease state and expressed through negative social reactions that may be perceived, experienced or internalized by WLWH” (27). For WLWH, this internalization leads to damaging psychological self-image, diminished self-value, and limited disclosure of health needs in clinical settings (27). In fact, a study noted that increased exposure to WLWH in clinical settings also increases healthcare provider-induced stigma in clinics in the US, particularly surrounding fertility intentions and pregnancy (26, 27). Combined with known disparities in education levels, insurance coverage, poverty, and access to healthcare (37), HIV-related stigma may contribute to ongoing and often hidden menstrual inequities. Studies exploring the overlap of HIV related stigma and menstrual stigma will be necessary to answer crucial questions towards achieving menstrual equity.

One specific facet of stigma that remains understudied is the role of blood stigma and its impact on menstrual health management. Studies on the degree of blood stigma in the HIV population are globally focused and centered around blood transfusion, postpartum bleeding and bleeding disorders (38–40). A review of blood stigma around transfusion access in Uganda noted that misconceptions about the transmission of HIV has hindered the ability for WLWH to acquire timely transfusion services and donations (41). Living with the combination of HIV and bleeding disorders, such as aplastic anemia or hemophilia, can exacerbate marginalization and psychosocial distress (38). The risk of perinatal HIV transmission contributes to stigma during pregnancy but whether concerns about infectivity—specifically through postpartum vaginal bleeding—is unexplored. One qualitative analysis of postpartum parents with HIV describes experiencing discrimination and obstetric violence by providers who feared exposure to bodily fluids (42). There are no follow up studies on how such institutional and interpersonal stigmas impact home management of postpartum and subsequent menstrual bleeding in this population in the United States.

## Discussion

In 2022, the World Health Organization called menstrual health and menstrual equity a matter of human rights and gender equity (33). Missing from this call are the voices of WLWH. Geographic, social and race-based disparities in HIV incidence intersect with many factors associated with menstrual equity (43, 44). The role of stigma, socioeconomic factors related to access to menstrual hygiene care, access to gynecologic care, and priorities of health providers may all contribute to a unique experience of menstruation for WLWH and contribute to menstrual inequity in the United States.

While menstrual needs and equity among specifically WLWH in low- and middle-income countries remain unexamined, evidence from studies on contraceptive use and discontinuation indicates that menstrual bleeding is a critical determinant of treatment success and adherence for WLWH. A 2010 qualitative study of 42 WLWH in South Africa revealed that contraception is often discontinued due to changes in bleeding patterns; interestingly, nearly 96% noted amenorrhea—an expected outcome from contraception use—as bothersome, reporting their desire for cycle return (45). A 2023 qualitative study of 17 South African WLWH who electively discontinued their intrauterine device most often cited increased bleeding after insertion as the reason for discontinuance (46). The ASPIRE Study exploring the adherence of the vaginal ring for HIV-1 prevention in four African countries noted that younger age, ring worries, condom use, and episodes of menstrual bleeding were associated with non-adherence (47). Owing to the paucity of research on menstrual irregularities and menstrual equity among women with HIV (WLWH) in global contexts, this study primarily relies on evidence from the United States and Canada. Additional research in low- and middle-income countries (LMICs) is imperative to inform context-specific menstrual health policies and support interventions.

Despite the complex interplay of social, cultural, economic, and legal determinants underlying menstrual inequities in the United States, existing legislative efforts have predominantly emphasized product accessibility, leaving other critical determinants insufficiently addressed (48, 49). In 2016, New York City became the first entity to pass several menstrual equity bills to improve access to period products in schools, correctional facilities, and housing service shelters (49). Since then, an additional sixty-two menstrual equity bills have been enacted across several states, aiming to eliminate menstrual sales taxes, expand access to free products, and mandate ingredient disclosure (49, 50). Currently, there are only two bills on menstrual equity at the federal level—one allowing menstrual products to be purchased with pre-tax dollars and the second requiring federal prisons to provide products (50). The more comprehensive Menstrual Equity for All Act, introduced by Congresswoman Grace Meng, continues to stall in Congress (50). Interestingly, despite the passage of these laws at the city and federal levels, vulnerable communities, such as those who are incarcerated (15) and those who are houseless (51)—two groups often overlapping with those living with HIV—continue to struggle with basic menstrual health and hygiene. Missing from these bills, however, is a focus on addressing menstrual stigma and menstrual health education for vulnerable communities, such as those living with HIV. A comprehensive menstrual health policy needs to go beyond simply access to supplies, especially when addressing the gaps in stigma, safety, and support for WLWH. Central to future policies is patient-centered research to improve the gap in menstrual health knowledge, to better measure menstrual burden, and address blood stigma, if any. Policies should include comprehensive menstrual and sexual health education in all institutions in a layered fashion starting at menarche. Additionally, “vaginal bleeding equity” should replace “menstrual equity” to be inclusive of all stages across reproductive life course and their specific supply and support needs (48).

Given the increased lifespan of WLWH and accumulated data on low HIV transmission risk with vaginal intercourse and pregnancy with ART use and viral load suppression, research on the intersection of stigma, perceptions of infectivity risk, and experiences with accessing menstrual products and care is necessary. Formative research in partnership with community members is necessary to learn about the lived experiences and drivers of decision making around menstrual equity and menstrual management in WLWH. Studies on the experiences of WLWH on discussing their menstrual health and needs in healthcare spaces is needed since many WLWH access comprehensive care—including gynecologic care—through HIV focused clinics (23). This focus on the intersection of menstrual equity and HIV care is essential to improve quality of life, support well-being, and affirm the dignity and human rights of all menstruators living with HIV.
